# A novel endoscopic finding of a scratch sign is useful for evaluating the *Helicobacter pylori* infection status

**DOI:** 10.1002/deo2.200

**Published:** 2022-12-22

**Authors:** Tomoyuki Yada, Yoshiyuki Itakura, Ryo Watanabe, Keita Odaka, Toyokazu Yagi, Yurika Ikegami, Katsunori Sekine, Naomi Uemura

**Affiliations:** ^1^ Division of Gastroenterology and Hepatology Kohnodai Hospital, National Center for Global Health and Medicine Chiba Japan; ^2^ Division of Gastroenterology Matsue Red Cross Hospital Shimane Japan

**Keywords:** endoscopic finding, esophagogastroduodenoscopy, gastritis, *Helicobacter pylori*, scratch sign

## Abstract

**Objectives:**

During esophagogastroduodenoscopy, a red linear scrape‐like appearance with white deposits sometimes appears on the gastric mucosa at the lower greater curvature of the gastric body, a finding we named the “scratch sign.” We aimed to clarify the clinical significance of this new endoscopic finding in the endoscopic evaluation of the *Helicobacter pylori* infection status.

**Methods:**

Among patients who underwent esophagogastroduodenoscopy at our hospital between October 2016 and June 2017, 437 patients were included in the study. We first examined the overall scratch sign positivity rate, and then this was compared according to the *H. pylori* infection status. Subsequently, other variables were compared and examined between the positive and negative scratch sign groups.

**Results:**

Overall, 437 patients were included in the analysis. The scratch sign was observed in 1.4% of 71 patients with current infections, 26.9% of 290 patients with past infections, and 31.6% of 76 uninfected patients. In the multivariate analysis, *H. pylori*‐negative, severe gastric mucosal atrophy, and acid secretion depressant were independent factors that significantly affected the appearance of the scratch sign.

**Conclusions:**

A novel endoscopic finding, the scratch sign, was found to be a good endoscopic predictor of *H. pylori*‐negative gastric mucosa. Furthermore, combined with atrophic changes and xanthomas that persisted after eradication, these findings were found to be useful in accurately diagnosing *H. pylori* past‐infected gastric mucosa endoscopically.

## INTRODUCTION

*Helicobacter pylori* infection, which is significantly involved in the pathogenesis of gastric cancer, induces chronic histological inflammatory changes in the gastric mucosa.^1^, ^2^ It was found that the prevalence of gastric cancer and metachronous gastric cancer after endoscopic resection could be reduced to approximately 50% by *H. pylori* eradication treatment,^3^, ^4^ and this treatment has been actively carried out. Conversely, the long‐term risk of gastric cancer persists even after *H. pylori* eradication; thus, continuous endoscopic surveillance is required even after eradication.^5^ Therefore, the number of encounters with the gastric mucosa after *H. pylori* eradication during routine endoscopic examinations is rapidly increasing. To evaluate gastric cancer risk based on endoscopic findings, it is important to first distinguish between *H. pylori*‐uninfected gastric mucosae with low gastric cancer risk and other *H. pylori*‐associated gastric mucosae (current and past infection). Subsequently, it is necessary to differentiate between the current and past *H. pylori* infections, and in cases of a current infection, patients should be guided to receive eradication treatment. The “Kyoto Classification of Gastritis,” published in 2014, is a method of evaluating the *H. pylori* infection status based on endoscopic findings and is now widely used in Japan.^6^ This classification divides patients into three groups: *H. pylori*‐uninfected patients (no gastritis), patients with current *H. pylori* infections (active gastritis), and patients with past *H. pylori* infections (inactive gastritis). However, although there are characteristic endoscopic findings for both *H. pylori*‐currently infected and uninfected gastric mucosae, there are few endoscopic findings that can be used to determine that the gastric mucosa is past‐infected because the past‐infected gastric mucosa is judged by the disappearance of active inflammatory findings.^7^ Among the endoscopic findings, map‐like redness is considered a characteristic feature of *H. pylori*‐past‐infected gastric mucosa; however, this feature is not always present.^8^, ^9^ Therefore, in routine endoscopic practice, the evaluation of the *H. pylori* infection status can sometimes be troublesome.^10^, ^11^

During esophagogastroduodenoscopy, when the duodenum is observed first and then the stomach is observed, a red linear scrape‐like appearance with white deposits sometimes appears on the gastric mucosa at the lower greater curvature of the gastric body. As we focused on this finding, we found it to be more common in *H. pylori*‐negative gastric mucosae than in mucosae that were currently infected by *H. pylori*; therefore, we named it the “scratch sign.”

In this study, we aimed to clarify the clinical significance of a new endoscopic finding, the scratch sign, in the endoscopic evaluation of the *H. pylori* infection status.

## METHODS

### Patients

Between October 2016 and June 2017, 2410 patients underwent esophagogastroduodenoscopy at our hospital. The scratch sign can easily be identified from a distant view if this finding is prominent. However, if not, it is necessary to recognize that this finding tend to occur in the lower greater curvature of the gastric body and to check the same area from a near view. Therefore, this study included patients examined by two experienced endoscopists (437 cases) who had been aware of this finding since these periods and had consciously taken the images. Patients with unknown *H. pylori* infection status, those who underwent intragastric observation before the insertion of an endoscope into the duodenum or those with insufficiently observed gastric mucosa owing to malignancy, active bleeding, accumulation of intragastric residues, or drug adhesion to the gastric mucosa, were excluded from the study.

This study was approved by the ethics review board at the National Center for Global Health and Medicine (NCGM‐G‐002467‐00). All clinical investigations were conducted per the ethical guidelines of the Declaration of Helsinki.

### Endoscopic procedure settings

Esophagogastroduodenoscopy was performed using the EVIS LUCERA ELITE system with high‐resolution scopes (GIF‐H290 and GIF‐H260 series, Olympus Medical Systems, Tokyo, Japan). We used a fixed structure‐enhancement setting and color tone. For endoscopic observation, the scope was inserted orally and the esophagus and duodenum were observed, followed by the observation of the stomach.

### Endoscopic evaluation of the gastric mucosa

**FIGURE 1:**
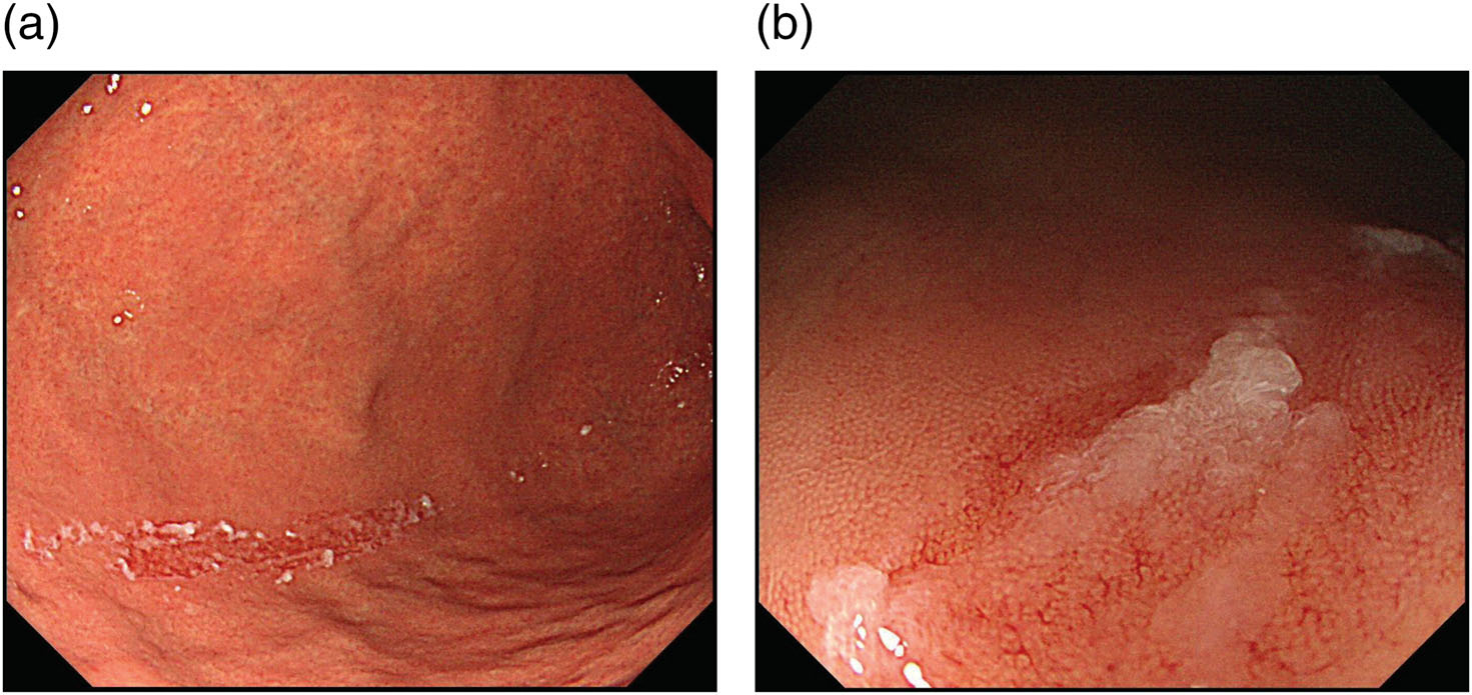
A typical image of the scratch sign: red lines with white adhesive materials traced as though scratching the gastric mucosa of the greater curvature of the stomach (a) distant view; (b) near view

Figure 1 shows a typical “scratch sign”; red linear scrape marks with white adherents observed in the longitudinal direction from the lower part of the stomach to the greater curvature of the gastric angle. Two expert endoscopists reviewed all target endoscopic images, and scratch sign positivity was defined as a clear scrape mark with a length of at least 5 mm.

The endoscopic evaluation of gastric mucosal atrophy was classified into no atrophy (C0), mild atrophy (C1–C2), moderate atrophy (C3–O1), and severe atrophy (O2–O3) using the Kimura–Takemoto classification.^12^ The Kyoto classification score was also assessed as follows: (1) atrophy—score 0 (Kimura–Takemoto classification C0, C1), score 1 (C2, C3), and score 2 (O1, O2, O3); (2) intestinal metaplasia—score 0 (none), score 1 (within the antrum), and score 2 (up to the corpus); (3) enlarged folds—score 0 (negative), score 1 (positive); (4) nodularity—score 0 (negative), score 1 (positive); and (5) diffuse redness—score 0 (negative), score 1 (mild), and score 2 (severe).

### Evaluation of the *H. pylori* infection status

The evaluation of the *H. pylori* infection status was performed via an endoscopic evaluation of gastric mucosal atrophy and a history of eradication treatment per the anamnesis, and at least one positive diagnostic test, such as the urea breath test (Otsuka Pharmaceutical Co., Ltd, Tokyo, Japan), rapid urease test (Otsuka Pharmaceutical Co., Ltd, Tokyo, Japan), *H. pylori* IgG E‐plate (Eiken Chemical Co., Ltd, Tokyo, Japan), *H. pylori* stool antigen test (Wakamoto Pharmaceutical Co., Ltd, Tokyo, Japan), as well as histological evaluation in gastric mucosal biopsy tissues. The cut‐off value for the *H. pylori* IgG E‐plate test was 10 U/ml (as specified by the manufacturer), but for 3.0–9.9 U/ml, we have added the urea breath test or *H. pylori* stool antigen test because of the gray zone that includes current and previous infections. When results were positive for one or more of the above diagnostic tools and endoscopic gastric mucosal atrophy, the patient was diagnosed with current *H. pylori* infection. When results were negative for one or more of the above diagnostic tools and there was no endoscopic gastric mucosal atrophy, the patient was identified as *H. pylori*‐uninfected. When results were negative for one or more of the above diagnostic tools and the patient had an eradication history and/or endoscopic gastric mucosal atrophy, the patient was diagnosed with a past *H. pylori* infection.

### Analysis method

The primary endpoint of this study was to evaluate the utility of the scratch sign in the endoscopic assessment of the *H. pylori* infection status. The secondary endpoints were the evaluation of factors influencing the appearance of the scratch sign and its association with map‐like redness.

For the above evaluation, we first examined the overall scratch sign positivity rate, which was compared according to the *H. pylori* infection status. Subsequently, age, sex, *H. pylori* infection status, degree of gastric mucosal atrophy, use of acid secretion inhibitor (proton pump inhibitor [PPI] or potassium‐competitive acid blocker [PCAB]), use of nonsteroidal anti‐inflammatory drugs, use of antithrombotic medication, and Kyoto classification score were compared and examined between the scratch sign‐positive and scratch sign‐negative groups. The *H. pylori* infection status and degree of gastric mucosal atrophy in the scratch sign‐positive and map‐like redness‐positive groups were also evaluated.

### Statistical analysis

Statistical analyses were performed using the chi‐square test or Fisher's exact test for categorical variables and the *t*‐test or the Mann–Whitney *U* test for continuous variables. Multivariate analyses were also performed via logistic regression to identify factors influencing the appearance of the scratch sign. Factors with a *p*‐value of <0.05 in the univariate analysis were used for the multivariate analysis. The threshold for statistical significance was set at *p* < 0.05. All statistical analyses were performed using EZR (Saitama Medical Center, Jichi Medical University, Saitama, Japan).

## RESULTS

Overall, 437 patients (226 males and 211 females with a median age of 72 years) were included in the analysis. Among them, 71 (16.2%) were currently infected with *H. pylori*, 290 (66.4%) had a past *H. pylori* infection, and 76 (17.4%) were not infected with *H. pylori*. The scratch sign was observed in 103 cases (23.6%; Table 1).

**TABLE 1:** Characteristics of the 437 patients included in the study

		Scratch sign		
	Total	Positive	Negative	*p*‐value
No. of patients	437	103	334	
Age, range	72 (28–95)	74 (28–85)	72 (28–95)	0.067
Sex, male/female	226/211	44/59	182/152	0.042
*Helicobacter pylori* status				<0.001
Current	71 (16.2%)	1	70	
Past	290 (66.4%)	78	212	
Uninfected	76 (17.4%)	24	52	
Gastric mucosal atrophy				<0.001
None	76 (17.4%)	24	52	
Mild	48 (11.0%)	16	32	
Moderate	202 (46.2%)	56	146	
Severe	111 (25.4%)	7	104	
PPI or PCAB (+)	114 (26.1%)	9 (8.7%)	105 (31.4%)	<0.001
NSAIDs (+)	15 (3.4%)	2 (1.9%)	13 (3.9%)	0.537
Antithrombotic agent (+)	55 (12.6%)	4 (3.9%)	51 (15.3%)	0.002

*Note*: Gastric mucosal atrophy was evaluated using the classification of Kimura and Takemoto (C0: none, C1–C2: mild, C3–O1: moderate, and O2–O3: severe).

Abbreviations: NSAIDs, nonsteroidal anti‐inflammatory drugs; PCAB, potassium‐competitive acid blocker; PPI, proton pump inhibitor.

### *H. pylori* infection status and the scratch sign

The scratch sign was observed in 1.4% of 71 currently infected patients, 26.9% of 290 patients with a past infection, and 31.6% of 76 uninfected patients (Table 1). The scratch sign positivity rate was significantly higher among uninfected patients and those with past *H. pylori* infection than among those with current *H. pylori* infection. No significant difference was found between patients with past *H. pylori* infection and uninfected patients. In addition, when the scratch sign was used to indicate the absence of *H. pylori* infection (past infection and non‐infection), the sensitivity was 0.27, the specificity was 0.98, and the positive predictive value was 0.99 (Table 2).

**TABLE 2:** Scratch sign used as an indicator of *Helicobacter pylori* negativity

Sensitivity	0.27
Specificity	0.98
Positive predictive value	0.99
Negative predictive value	0.21

*Note*: *H. pylori*‐negative includes past infection and non‐infection.

### Comparison by the presence or absence of the scratch sign

Comparisons were made between the two groups of 103 patients with and 334 patients without the scratch sign via univariate analyses. Significant differences were found in the following parameters: female sex (*p* = 0.042), *H. pylori* infection status (*p* < 0.001), degree of gastric mucosal atrophy (*p* < 0.001), acid secretion depressant treatment (*p* < 0.001), and antithrombotic treatment (*p* = 0.002; Table 1). In other words, the scratch sign was more common among females and *H. pylori*‐negative individuals (past infection and non‐infection) than patients with severe gastric mucosal atrophy and those who consumed acid secretion depressants or antithrombotic agents. In the multivariate analysis, *H. pylori*‐negative (OR 28.1 [95% CI 3.80–208.00]), severe gastric mucosal atrophy (OR 0.16 [95% CI 0.07–0.36]), and acid secretion depressant (OR 0.21 [95% CI 0.10–0.45]) were significant and independent factors affecting the appearance of the scratch sign (Table 3). The scratch sign‐positive group had significantly lower Kyoto classification scores for atrophy (*p* = 0.005), intestinal metaplasia (*p* = 0.016), enlarged folds (*p* = 0.001), and diffuse redness (*p* < 0.001). Moreover, the scratch sign‐positive group had a significantly lower total Kyoto classification score than the scratch sign‐negative group (*p* < 0.001; Table 4).

**TABLE 3:** Multivariate logistic regression analysis of subjects with the scratch sign

Variable	Odds ratio	95% Confidence interval	*p*‐Value
*Helicobacter pylori* negative	28.1	3.80–208.00	0.001
Female	1.55	0.95–2.53	0.082
Severe mucosal atrophy	0.16	0.07–0.36	<0.001
PPI or PCAB	0.21	0.10–0.45	<0.001
Antithrombotic agent	0.53	0.17–1.64	0.271

Abbreviations: PPI, proton pump inhibitor; PCAB, potassium‐competitive acid blocker.

**TABLE 4:** Comparison of Kyoto classification scores according to the presence of scratch sign

Scratch sign			
	Positive	Negative	*p*‐value
Atrophy	1.14 ± 0.77	1.37 ± 0.74	0.005
Intestinal metaplasia	0.54 ± 0.64	0.76 ± 0.83	0.016
Enlarged folds	0.02 ± 0.14	0.13 ± 0.34	0.001
Nodularity	0.00 ± 0.00	0.01 ± 0.08	0.432
Diffuse redness	0.00 ± 0.00	0.39 ± 0.77	<0.001
Total score	1.70 ± 1.30	2.65 ± 1.75	<0.001

*Note*: Data show mean ± standard deviation.

### Comparison of scratch sign‐positive and map‐like redness‐positive patients

Although the scratch sign was characteristic of patients with past *H. pylori* infection and uninfected patients, map‐like redness was specific to past *H. pylori* infection and in 53 (18.3%) of the 290 past‐infected cases. In gastric mucosal atrophy, the majority of patients had no atrophy or mild atrophy for the scratch sign, whereas all patients had moderate or severe atrophy for the map‐like redness (Table 5).

**TABLE 5:** Comparison of scratch sign‐positive patients to map‐like redness‐positive patients

	Scratch sign (+) *n* = 103	Map‐like redness (+) *n* = 53
*Helicobacter pylori* status		
Current (*n* = 71)	1 (1.4%)	0 (0.0%)
Past (*n* = 290)	78 (26.9%)	53 (18.3%)
Uninfected (*n* = 76)	24 (31.6%)	0 (0.0%)
Gastric mucosal atrophy		
None (*n* = 76)	24 (31.6%)	0 (0.0%)
Mild (*n* = 48)	16 (33.3%)	0 (0.0%)
Moderate (*n* = 202)	56 (27.7%)	20 (9.9%)
Severe (*n* = 111)	7 (6.3%)	33 (29.7%)

### Histopathological findings

**FIGURE 2:**
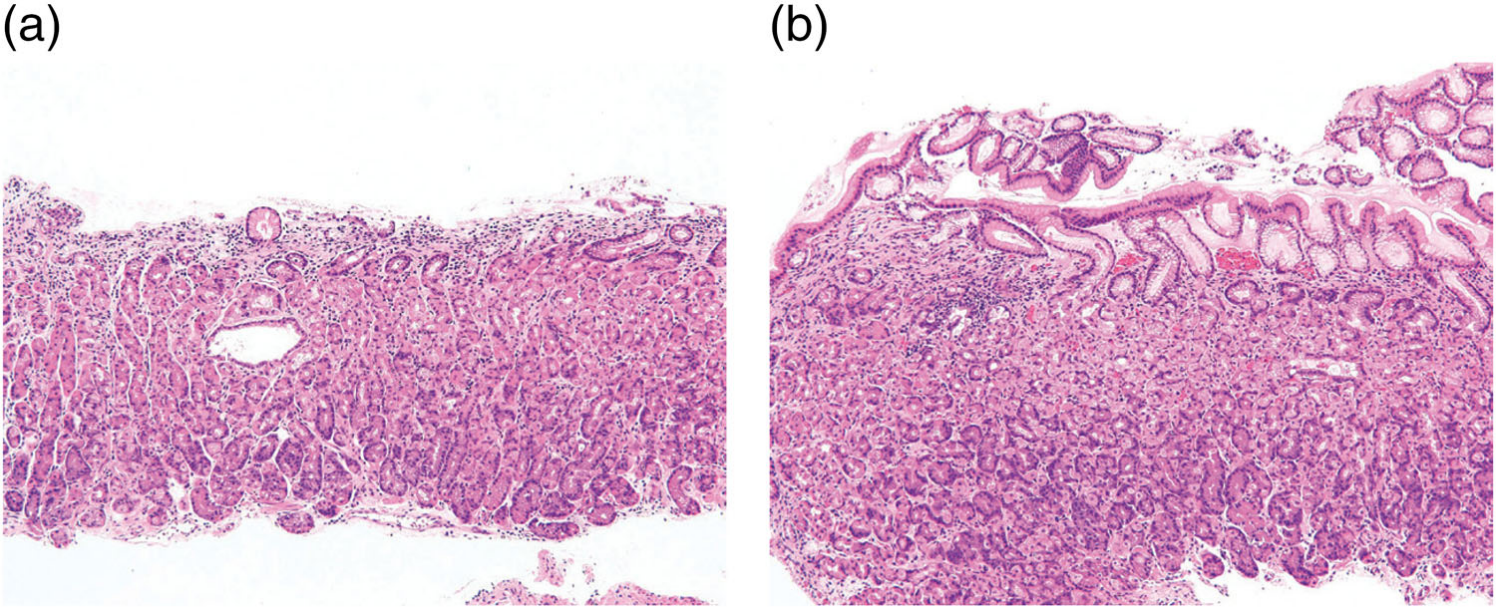
Comparison of the scratch sign‐positive and ‐negative areas in biopsy tissue obtained from the same patient. (a) Scratch sign‐positive area (hematoxylin and eosin stain ×100); (b) scratch sign‐negative area (hematoxylin and eosin stain ×100)

Biopsy tissue samples were collected from the scratch sign‐positive and scratch sign‐negative areas in some patients. Histopathologically, the foveolar epithelium of the superficial layer of the mucosa was detached and missing in the areas with a positive scratch sign (Figure 2).

## DISCUSSION

This study evaluated the utility of a novel endoscopic finding, the scratch sign, in the endoscopic evaluation of the *H. pylori* infection status. The scratch sign was identified as a common finding in *H. pylori*‐negative individuals. This may be an important indicator for endoscopic evaluation of the *H. pylori* infection status in routine practice.

*H. pylori* infection induces chronic active gastritis.^1^ The histopathological classification of gastritis includes the Sydney System and the Updated Sydney System^13^, ^14^; however, it has not been widely used in routine clinical practice due to the low concordance rate among pathologists and reproducibility issues, and also because it does not directly reflect the gastric cancer risk. Hence, the operative link for gastritis assessment and operative link on gastric intestinal metaplasia sssessment have been identified as more convenient and risk‐conscious classification systems for gastric cancer, which have become widespread mainly in Western countries.^15^, ^16^ However, the above method requires multiple biopsies, and the bleeding risk poses a problem in the elderly and patients taking antithrombotic drugs.^17^, ^18^

Conversely, the Kyoto classification of gastritis has been proposed and widely used in Japan, in which gastritis is evaluated based on 19 characteristic endoscopic findings of *H. pylori* infection rather than biopsies, and classified into current infections, past infections, and uninfected cases.^6^ In the same classification, five of the^19^ evaluation items (atrophy, intestinal metaplasia, enlarged folds, nodularity, and diffuse redness) are selected and scored to stratify the risk of gastric cancer.^19^, ^20^, ^21^ More recently, not only has white‐light imaging been reported for the evaluation of gastritis but the utility image‐enhanced endoscopy (IEE) has also been reported.^22^, ^23^, ^24^ In some cases, a minor scratch sign is more easily identified on narrow band imaging (NBI). The effects of IEE, including NBI, on the visibility of scratch signs should be further investigated.

Based on the Kyoto classification of gastritis, map‐like redness is an important and characteristic finding observed only in *H. pylori* past‐infected gastric mucosa; however, its positivity rate is estimated to be 10%–30%.^6,8,9^ In this study, map‐like redness was observed in 18.3% of cases of past‐infected cases, which was the same as the frequency reported. Map‐like redness tends to appear in patients with severe gastric mucosal atrophy due to intestinal epithelialization. In contrast, the scratch sign tends to appear in patients with no or mild gastric mucosal atrophy. In fact, in the present study,^6^ (11.3%) of the 53 past‐infected patients with map‐like redness were found to have the scratch sign, whereas 72 (30.4%) of the 237 past‐infected patients without map‐like redness were found to have the scratch sign (data not shown). The scratch sign is an *H. pylori*‐negative finding that includes past‐infected and uninfected patients; however, past‐infected and uninfected patients can easily be differentiated via the presence of atrophy or xanthoma.^10^ Therefore, when atrophy or xanthoma is present, the presence of map‐like redness is a diagnostic feature of past‐infected gastric mucosa; however, in cases of mild atrophy with no map‐like redness, the presence of the scratch sign is a diagnostic feature of past‐infected gastric mucosa.

**FIGURE 3:**
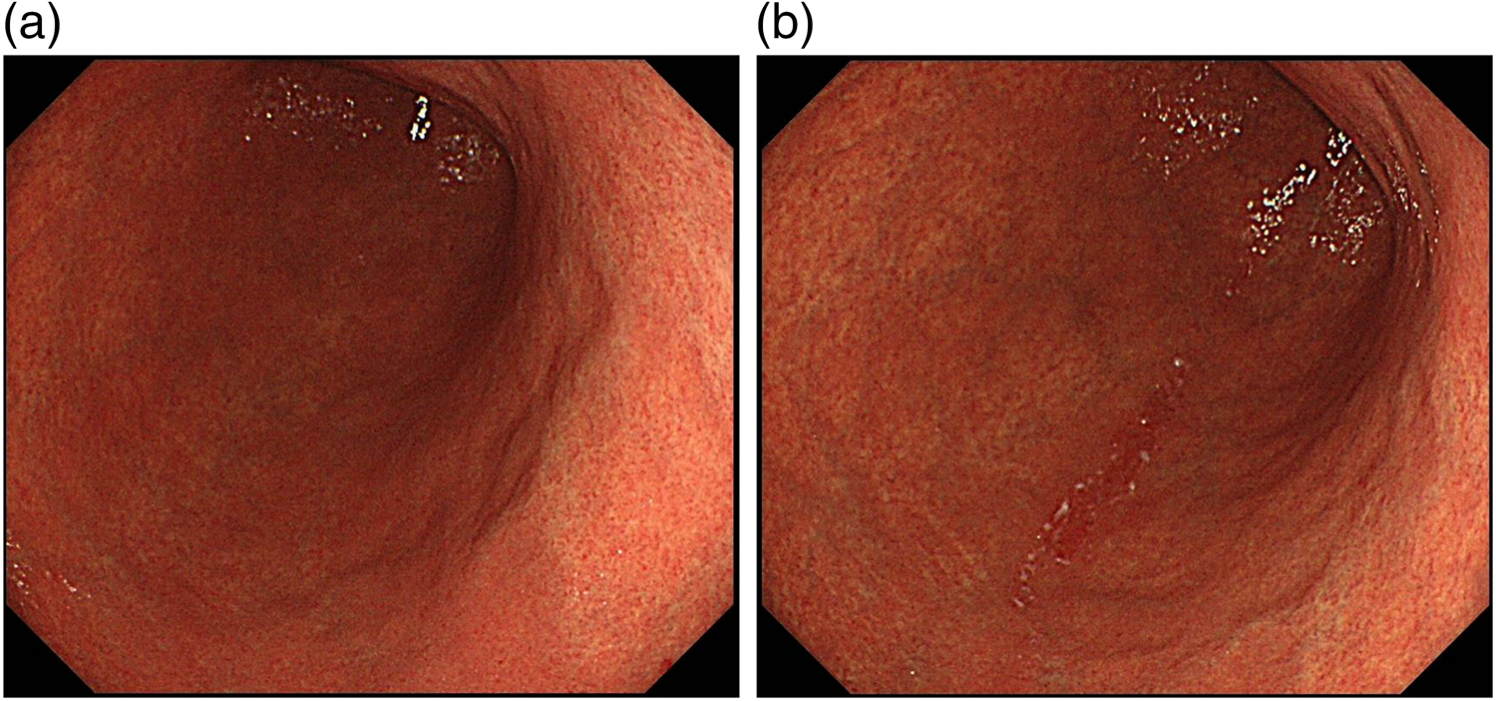
(a) Before insertion into the duodenum; the scratch sign is negative, (b) after insertion into the duodenum; the scratch sign is positive

The scratch sign is not observed only via intragastric manipulation but is observed after stretching the stomach during duodenal observation, and the mechanism of occurrence is thought to be mechanical stimulation associated with the pressure of the scope (Figure 3). In the denuded gastric mucosa where the foveolar epithelium is detached and lost by the scope, the vascular architecture of the mucosal lamina propria could be observed through the mucosa, which is thought to be visible as an erythematous area. We speculate that the mucus gel layer contributes to this phenomenon. The gastric mucus gel layer is preserved in *H. pylori*‐uninfected and past‐infected patients, whereas the layer structure is disturbed in currently infected patients; in addition, the mucus gel layer becomes thinner in intestinal metaplasia.^25^ The gastric mucus gel also showed decreased viscosity at increased pH, such as what happens during PPI or PCAB administration, and it is known that viscosity decreases even through *H. pylori* infection.^26^, ^27^ In this study, the scratch sign was more likely to occur in *H. pylori*‐negative patients and less likely to occur in patients with severe gastric mucosal atrophy and those taking acid secretion inhibitors. Therefore, the appearance of the scratch sign may require a situation in which the mucus gel layer is thick and viscosity is maintained. The pathogenesis of the scratch sign is a subject for future research.

This study had several limitations. First, the present study is a preliminary examination of a small number of patients examined by a limited number of endoscopists to understand the significance of the scratch sign noticed for the first time. To establish the usefulness of the scratch sign, prospective clinical studies, including the Kyoto classification of gastritis, are required in the future. Second, it used only oral endoscopes measuring approximately 10 mm and was not verified for thin endoscopes, such as transnasal endoscopes. Allegedly, the scratch sign was recognized even with the transnasal endoscope; however, the frequency should be examined in the future. Third, we did not rigorously use multiple methods to diagnose *H. pylori* infection.

In conclusion, a novel endoscopic finding, the scratch sign, was found to be a good endoscopic predictor of *H. pylori*‐negative gastric mucosae. Furthermore, combined with atrophic changes and xanthomas that persisted after the eradication of *H. pylori*, these findings were found to be useful in accurately identifying *H. pylori*‐past‐infected gastric mucosae via endoscopy.

## CONFLICTS OF INTEREST

None.
